# Inhibiting the proton pump: mechanisms, benefits, harms, and questions

**DOI:** 10.1186/s12916-016-0724-1

**Published:** 2016-11-09

**Authors:** Jeffrey K. Aronson

**Affiliations:** Centre for Evidence-Based Medicine, Nuffield Department of Primary Care Health Sciences, University of Oxford, Radcliffe Observatory Quarter, Oxford, OX2 6GG UK

**Keywords:** Proton pump inhibitors, Benefits, Harms, Adverse drug reactions, Drug–drug interactions

## Abstract

Inhibition of the H^+^/K^+^-adenosine triphosphatase (the proton pump) is the final common mechanistic pathway in reducing gastric acid secretion pharmacologically. Proton pump inhibitors are widely used in upper gastrointestinal diseases, including gastric and duodenal ulcers, eradication of *Helicobacter pylori* in combination with antibiotics, gastroesophageal reflux disease, Zollinger–Ellison syndrome, eosinophilic esophagitis, and prevention of non-steroidal anti-inflammatory drug-induced peptic ulceration. Reviewing their benefits and harms in *BMC Medicine*, Scarpignato et al. report effectiveness in these conditions, and harms that are generally mild and uncommon (1–3 %). Serious adverse reactions, such as tubulointerstitial nephritis, are rare. However, the risks of gastric and pancreatic cancer are unclear. Drug–drug interactions can occur through effects on P glycoprotein and cytochrome P450 (CYP) isoenzymes. Several questions remain. Do all proton pump inhibitors carry the same risks of serious adverse reactions? Which individuals are most susceptible? What are the time courses of individual reactions? What monitoring strategies are best? New drugs for the same indications continue to emerge, including potassium-competitive acid blockers, inhibitors of transient lower esophageal sphincter relaxation, serotonergic agents/prokinetics, mucosal protectants, histamine H_3_ receptor agonists, anti-gastrin agents, and esophageal pain modulators. Their benefit to harm balance remains to be discovered.

Please see related article: https://bmcmedicine.biomedcentral.com/articles/10.1186/s12916-016-0718-z

## Background

When I was a medical student in the 1960s both physicians and surgeons managed peptic ulcer disease. Medical therapy was largely unsatisfactory, the available medicines being poorly effective. When it failed, or when an ulcer bled or perforated, surgical intervention was often required. The techniques used included partial gastrectomy with anastomosis to the duodenum (Billroth I), gastrectomy with gastrojejunostomy (Billroth II), Roux-en-Y bypass [1], and highly-selective vagotomy with or without pyloroplasty [2, 3]. The results were often good, but at the cost of unwanted effects [4, 5], including dumping syndrome [6], stomal ulceration [7], gastrojejunocolic fistulae [8], and a risk of cancer in the gastric stump [9].

However, in the 1970s more effective medicines and new therapeutic approaches started to emerge. For example, high doses of antacids, apart from providing symptomatic relief, could be curative [10, 11]. Similar recurrence rates were observed after the end of therapy with both antacids and histamine H_2_ receptor antagonists [12], and long-term therapy with either treatment maintained healing equally well [13].

**Fig. 1:**
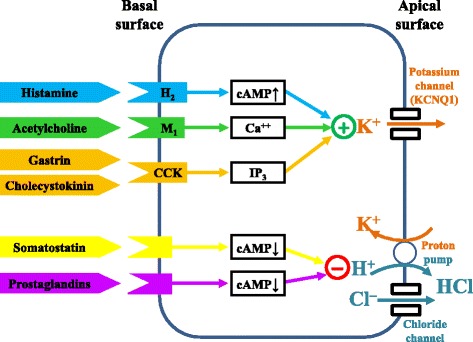
A simplified description of the physiology of gastric acid secretion; other compounds involved in its regulation, not shown, include ghrelin, glutamate, pituitary adenylase cyclase-activating peptide (PACAP), and serotonin (5HT)

**Fig. 2:**
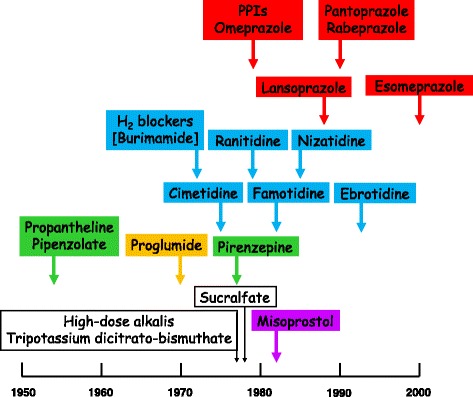
Some drugs used to modulate gastric acid secretion and their times of introduction (earliest papers listed in PubMed); some are no longer in use; the color coding corresponds to that in Fig. 1

Nowadays, although surgery is still sometimes performed, peptic ulceration and its complications are largely managed by physicians, whether by endoscopic or pharmacological means. Fiber-optic endoscopy came of age in the 1970s [14] and elucidation of the physiology of gastric acid secretion (Fig. 1) [15] led to the development of medicines that reduce gastric acid secretion (Fig. 2), such as anticholinergic drugs selective for muscarinic M_1_ receptors (pirenzepine), histamine H_2_ receptor antagonists, and prostaglandins, the last specifically used in preventing peptic ulceration in patients taking non-steroidal anti-inflammatory drugs (NSAIDs).

The H^+^/K^+^-adenosine triphosphatase (the proton pump) in the apical surfaces of gastric parietal cells is the final common pathway for medicines with different mechanisms of action that alter gastric acid secretion. This observation led to the development of a range of substituted benzimidazoles that inhibit the pump, which have come to be known as proton pump inhibitors (PPIs), of which omeprazole was the first to be developed for clinical use [16]. Recently, newer reversible PPI inhibitors, potassium-competitive acid blockers such as vonoprazan, have also become available [17].

## Proton pump inhibitors: benefits

Members of la Società Italiana di Farmacologia (SIF), l’Associazione Italiana dei Gastroenterologi Ospedalieri (AIGO), and la Federazione Italiana dei Medici di Medicina Generale (FIMMG), with the aid of external consultants, have prepared a lengthy position paper, containing nearly 500 references, in which they outline the major benefits and harms of PPIs [18]. It is not a systematic review, although systematic reviews are cited, but an extensive scoping review, containing 13 essays on the uses of PPIs in a range of conditions, with an accompanying summary table of conclusions, and an essay on the harms that they can cause, with two accompanying tables listing gastrointestinal and other harms.

Box 1 gives a summary of the benefits, using the headings listed in the paper and quoting directly from the text.

## Proton pump inhibitors: harms and questions

The authors of the position paper report that harms attributable to PPIs occur in 1–3 % of cases and are for the most part minor, and that serious adverse reactions, such as tubulointerstitial nephritis, are rare [18]. They stress that PPIs are often prescribed inappropriately, especially in elderly people, but that the benefit to harm balance is good in patients in whom treatment is appropriate. They list the most common untoward effects as including headaches, nausea, abdominal pain, constipation, flatulence, diarrhea, rashes, and dizziness.

However, some important harms need to be recognized. Take, for example, the risk of intestinal *Clostridium difficile* infection. In a study of 54,957 patients taking PPIs in Australia, Canada, Japan, and Korea, the pooled adjusted sequence ratio of the risk of *C. difficile* infection was 2.40 (95 % confidence interval [CI]: 1.88, 3.05); the risk was detectable within the first 2 weeks of treatment and did not vary by individual PPI [19]. In 1187 inpatients in Canada who were given antibiotics, the risk of *C. difficile* infection was increased, with an odds ratio of 2.1 (95 % CI: 1.2, 3.5), and was associated with female sex and prior renal insufficiency; histamine H_2_ receptor antagonists did not increase the risk [20]. Conversely, in one systematic review and meta-analysis there was an increased risk associated with histamine H_2_ receptor antagonists [21], even though a year earlier the same authors had found only very low-quality evidence for an association between PPI use and *C. difficile* infection, with no support for a cause-and-effect relationship [22]. In another study there were increased risks with both PPIs and H_2_ receptor antagonists, the risk being higher with the former; diabetes mellitus was an added susceptibility factor [23]. An increased risk of gut *Candida* infections has also been suggested [24]. All this raises the question of whether one should withhold PPIs and histamine receptor antagonists when starting antibiotic therapy, particularly for patients in hospital. At present, one would recommend doing so, but we do not know what the balance of benefit to harm is, and the literature on this important topic is disparate and confusing.

Another harm to consider is the risk of gastric carcinoma during long-term PPI therapy. The early fears that reduced gastric acid secretion and the associated hypergastrinemia might induce this complication and limit the use of PPIs have not been allayed. When I searched the World Health Organization’s VigiBase database of suspected adverse reactions, I found significant disproportionalities for three of the five currently marketed PPIs, with Information Criterion (IC) values ranging from 1.77 to 2.58. Recent systematic reviews also suggest an association [25, 26], and this problem needs further study. The risk of pancreatic carcinoma, which is currently increasing in general [27], also requires clarification.

Other questions about harms due to PPIs remain to be answered. Do all PPIs carry the same risks of serious adverse reactions? Which individuals are most susceptible? What are the time courses of individual reactions? What monitoring strategies are best? How often do important drug–drug interactions occur, through effects on P glycoprotein and cytochrome P450 (CYP) isoenzymes such as CYP3A4 and CYP2C19, and are some PPIs less likely to take part in them? Interactions with thienopyridines such as clopidogrel, antiretroviral drugs, and anticancer drugs have recently been highlighted [28].

Finally, we await information on the effects of newer compounds with different mechanisms of action, including potassium-competitive acid blockers, inhibitors of transient lower esophageal sphincter relaxation, serotonergic agents/prokinetics, mucosal protectants, histamine H_3_ receptor agonists, anti-gastrin agents, and esophageal pain modulators [29, 30].

PPIs are not the end of the story.

## Box 1

1. Gastroesophageal reflux disease (GERD, including non-erosive reflux esophagitis and Barrett’s esophagus): “the mainstay of medical treatment”

2. Eosinophilic esophagitis: “a first-line treatment”

3. *Helicobacter pylori* eradication and peptic ulcer disease: “a key component of current regimens”

4. Zollinger–Ellison syndrome: “the drugs of choice”

5. Stress ulcer prophylaxis: “the drugs of choice for acid suppression”

6. Dyspepsia: “treatment should be attempted in patients with persisting symptoms despite successful eradication or naïve-uninfected patients with epigastric pain syndrome”

7. NSAID-associated gastrointestinal (GI) symptoms and lesions: “standard doses indicated … more effective than H_2_ receptor antagonists”

8. Corticosteroid use: “not routinely indicated”

9. Anti-platelet or anticoagulant therapy: “standard dose therapy advised”

10. Peptic ulcer bleeding: “endoscopy is the mainstay of treatment; PPI therapy [thereafter] reduces the risk of re-bleeding, requirement for surgery, and mortality in high-risk patients”

11. Patients with cancer: “could be indicated to treat or/and prevent [symptomatic] chemotherapy-induced GERD and gastro-duodenal ulceration; patients with GI mucositis or dysphagia might also benefit” [poor-quality evidence]

12. Cirrhosis: “not justified”

13. Pancreatic disease: “not recommended”
